# Dose-Dependent Effect of Hyperoside on the Physicochemical and Gel Properties of Porcine Myofibrillar Proteins at Different NaCl Concentrations under Oxidative Stress

**DOI:** 10.3390/foods12081684

**Published:** 2023-04-18

**Authors:** Xiuyun Guo, Shuangyi Xu, Xiangren Meng, Zengqi Peng

**Affiliations:** 1 School of Turism and Cuisine, Yangzhou University, Yangzhou 225127, China; 007840@yzu.edu.cn (X.G.);; 2 College of Food Science and Technology, Nanjing Agricultural University, Nanjing 210095, China

**Keywords:** myofibrillar proteins, HYP, oxidative characteristics, gel properties

## Abstract

The effects of HYP (10, 50, and 250 μM/g protein) on the physicochemical and gel properties of myofibrillar proteins (MPs) at different NaCl concentrations under oxidative stress were explored. The incorporation of HYP significantly reduced carbonyl content and decreased the loss of free amine groups in a dose-dependent manner, regardless of NaCl concentration. In addition, HYP induced a dose-dependent decrement in total sulfhydryl content regardless of NaCl concentration, which might result from the formation of thiol-quinone adducts via Michael addition. The surface hydrophobicity was significantly increased with HYP addition. Nevertheless, compared with samples treated with 50 μM/g HYP, 250 μM/g HYP caused a significant decrease in surface hydrophobicity, which might be due to the increase in the extent of MPs unfolding and the concomitant aggregation of MPs by hydrophobic interaction. Furthermore, HYP also showed a dose-dependent increment in the water-holding capacity (WHC) and gel strength of MPs gels, which might be due to more orderly crosslinks via fibrous filaments at 0.2 M NaCl and more regular and lamellar structures with smaller and more homogeneous pores at 0.6 M NaCl. In summary, HYP reduced the oxidation-mediated changes of physicochemical characteristics, preventing the oxidative damage of MPs and reinforcing the ordered crosslinks of MPs–MPs and MPs–HYP during thermal gelation, ultimately resulting in a better gel quality. These results provide a theoretical support for the practical application of HYP as a natural antioxidant in gel-type meat products.

## 1. Introduction

Myofibrillar proteins (MPs) are the major proteins in muscle and their structural changes during heating provide a good texture and water-holding capacity (WHC) of meat products [1]. However, the active groups of the amino acid residues of MPs are easily attacked by the free radicals generated during meat processing or storage, which prevents the formation of covalent crosslinking of MPs during heating. In addition, the oxidative modifications cause the unfolding of MPs, leading to protein aggregation, which has a negative effect on the gel properties of MPs [2]. As a consequence, regulating MPs oxidation remains is of vital importance [3].

Currently, the addition of antioxidants is one of the typical methods used to prevent protein and lipid oxidation in meat products, resulting from their natural capacity for scavenging reactive oxygen species (ROS) [4]. The synthetic antioxidants, such as butylated hydroxyanisole and butylated hydroxytoluene, have been widely used in meat products due to their effectiveness against oxidation reactions. Nevertheless, the long-term consumption of synthetic antioxidants may relate to potential adverse health risks, such as carcinogenic risk, which results in the increased demand for natural antioxidants [5].

The natural antioxidants extracted from spices and herbs are widely used in meat products to prevent protein and lipid oxidation, which has been ascribed to their radical-scavenging and metal-chelating capacity [6,7,8]. Natural antioxidants, such as polyphenols, contain multiple hydroxyl and carboxyl groups, which can donate hydrogen protons and electrons to stabilize free radicals [9]. Nevertheless, the efficacy of natural antioxidants in preventing meat from oxidative damage considerably depends on the components of the extracts and meat matrix [10,11]. Besides their antioxidative function, some natural antioxidants can also improve the physicochemical properties of meat proteins and the sensory characteristics of meat products [12]. To date, polyphenolic compounds have been identified as the main effective components of these natural antioxidants; by-products of agriculture rich in polyphenols are usually recognized as potential sources of natural antioxidants due to their antioxidative and economic efficiency [6,13].

Zanthoxylum bungeanum Maxim., native to Eastern China, is commonly used to give special flavor to meat products. As a by-product of Zanthoxylum bungeanum, Zanthoxylum bungeanum Maxim. leaves (ZML) have been proven to be rich in polyphenols [13,14]. Li et al. found that the addition of ZML extract decreased lipid oxidation both in the dorsal and ventral muscle during the processing of salted fish [14]. As the major bioactive component of polyphenols in ZML, hyperoside (HYP, quercetin-3-*O*-β-D-galactoside) has attracted attention due to its various biological effects, such as anti-inflammatory, anticancer, and antioxidant activities [15]. In the study of Li et al., HYP exhibited a strong inhibitory effect on lipid oxidation by increasing the activity of antioxidant enzymes in salted silver carp. In addition, HYP expressed a high total antioxidant capacity and DPPH free radical scavenging activity [16]. Therefore, HYP has the potential to control protein oxidation. Nevertheless, no experimental studies have examined the effect of HYP on protein oxidation in meat and meat products.

Hence, the purpose of the present work was to explore whether HYP can inhibit the oxidation of MPs. A Fenton oxidation system was employed to induce MPs oxidation. The physicochemical and gel properties of porcine MPs under oxidation conditions were investigated. The results may provide a theoretical basis and reference for the comprehensive application of natural antioxidants containing HYP in meat products.

## 2. Materials and Methods

### 2.1. Materials

Fresh *Longissimus dorsi* from three pork carcasses were purchased from the local Yonghui market (Yangzhou, China). HYP and all other chemicals were of analytical-reagent grade and bought from Aladdin Co., Ltd. (Shanghai, China).

### 2.2. MPs Extraction

MPs were prepared in accordance our previous study [17]. Briefly, 100 g of pork was homogenized with 400 mL extracting solution containing 10 mM ethylenediamine tetraacetic acid (EDTA) and 100 mM Tris (pH 8.3) using a blender (Midea, Foshan, Guangdong, China). Then, the homogenate was centrifuged at 1500× *g* for 10 min at 4 °C using a high-speed centrifuge (Thermo Scientific SL8, Waltham, MA, USA). The precipitate was mixed three times with 4 volumes of buffer solution containing 100 mM potassium chloride (KCl), 2 mM magnesium chloride (MgCl_2_), 1 mM glycol-bis-(2-aminoethylether)-N, N, N′, N′-tetraacetic acid (EGTA), and 20 mM K_2_HPO_4_/KH_2_PO_4_ (pH 7.0). The mixture was filtered through two layers of gauze to remove residual connective tissue, and then centrifuged at 1500× *g* for 20 min at 4 °C. After centrifugation, the sediment was obtained and mixed with 4 volumes of distilled water. Once again, the mixture was centrifuged at 1500× *g* for 20 min at 4 °C, and the sediment was collected as MPs. The Biuret method was used to determine MPs concentration [18].

### 2.3. Preparation of Oxidative MPs Samples

A Fenton oxidation system was established as previously described by Huang et al. [8]. MPs samples (final protein concentration, 30 mg/mL) were prepared with eight treatments containing different concentrations of NaCl (0.2 and 0.6 M, 20 mM PBS, pH 6.5) and HYP (0, 10, 50, 250 uM/g MPs). Samples were oxidized using 1 mM H_2_O_2_, 10 μM FeCl_3_, and 100 μM ascorbic acid in the dark for 24 h at 4 °C. EDTA (final concentration, 1 mM) was applied to stop the reaction. Non-oxidized MPs at 0.6 M NaCl were treated as the blank.

### 2.4. Determination of Carbonyls

The method described by Zhou et al. [19] was used to measure carbonyl content. In brief, 1 mL MPs suspension in 20 mM PBS (protein concentration, 5 mg/mL, pH 6.5) was mixed with 4 volumes of 0.2% 2,4-dinitrophenylhydrazine (DNPH) or 2 M HCl (set as control) and reacted in the dark for 1 h at ambient temperature (25 °C). The mixtures were mixed with 4 volumes of 20% trichloroacetic acid (TCA) and centrifuged at 2000× *g* for 15 min at 4 °C. Then, the precipitate was mixed three times by 6 mL ethanol/ethyl acetate, followed by centrifugation at 2000× *g* for 10 min at 4 °C. The sediment was obtained and redissolved using 6 M guanidine hydrochloride. The absorbance at 370 nm was recorded to calculate the carbonyl content with the absorption coefficient of 21 mM^−1^ cm^−1^.

### 2.5. Determination of Free Amines Level

The method shown in the study of Zhao et al. [20] was used to analyze free amines levels with slight modification. Briefly, 200 μL of 4 mg/mL MPs was mixed with 2 mL of 1% SDS and 1 mL of 0.01% 2,4,6-trinitrobenzenesulfonic acid (TNBS). After reaction for 30 min at 50 °C (water bath), 2 mL of 0.1 M Na_2_SO_3_ was added to terminate the reaction. The absorbances at 420 nm of samples and L-leucine (set as the standard curve) were measured to calculate the free amine content of MPs.

### 2.6. Determination of Total Sulphydryl (SH) Content

The method described by Ellman [21] was used to measure SH content. Briefly, 20 μL 5,5′-dithiobis (2-nitrobenzoic acid) (DTNB) and 2.25 mL of 8 M urea were added to 0.75 mL of 4 mg/mL MPs (20 mM PBS, pH 6.5), followed by the incubation at 25 °C for 5 min. The content of SH was determined at 412 nm using a SpectraMax M3 microplate reader (Molecular Devices Ltd., Sunnyvale, CA, USA) with a molar extinction coefficient of 13,600 M^−1^ cm^−1^.

### 2.7. Determination of Surface Hydrophobicity

Bromophenol blue (BPB), which can bind to hydrophobic sites on proteins by hydrophobic interaction, was used to measure the surface hydrophobicity of MPs according to the method described in the study of Huang et al. [22]. Briefly, 200 μL of 1 mg/mL BPB was added to 1 mL of 5 mg/mL MPs and incubated at 25 °C for 10 min. Then, the mixture was centrifuged at 6000× *g* for 15 min at 4 °C and the supernatant was obtained. Ultimately, the samples were diluted 10 times. The absorbance values of the samples and PBS buffer (blank) were recorded at 595 nm. The surface hydrophobicity of MPs was expressed as the amount of BPB bound (μg), the calculation equation was as follows:

BPB bound (μg) = 200 μg × (A_595Blank_ − A_595sample_)/A_0_

### 2.8. Gel Strength and Water Holding Capacity (WHC)

In total, 10 mL of 30 mg/mL MPs was heated in a water bath from 25 °C to 85 °C at 2 °C/min and kept at 85 °C for 20 min. 

The gel strength was examined on the basis of the method described in the previous study [3] using a TAXT2i texture analyzer. 

WHC was also measured on the basis of the method described as follows: 5.0 g of MPs gels was centrifuged at 5000× *g* for 15 min at 4 °C, and the sediment was collected. The weight of the sediment was expressed as W.

WHC (%) = W/5 × 100

### 2.9. Cryo-Scanning Electron Microscopy (Cryo-SEM)

Images of the samples were acquired through cryo-SEM (Hitachi SU8010, Quorum PP3010T, TKY, JPN) on the basis of the method described in the study of Guo et al. [17]. Gels were prepared for cryo-SEM by sectioning a small sample (2 mm × 2 mm × 5 mm) and mounting it in a cylindrical plug of a copper sample holder such that a portion of the specimen protruded above the face of the holder to facilitate freeze fracture. Subsequently, the samples were rapidly frozen using liquid nitrogen at the temperature of −196 °C for 2 min. These specimens were then transferred to the preparation chamber cold stage and fractured, followed by sublimating at −70 °C for approximately 15 min under controlled vacuum conditions and sputtering with platinum at 10 mA for 60 s. Images were acquired at a 3 kV accelerating voltage.

### 2.10. Statistical Analysis

The data were analyzed using a statistical analysis system (SAS Institute Inc., Cary, NC, USA) for Duncan’s multiple range test and one-way ANOVA with a significance level of *p* < 0.05. The images were produced by OriginPro 12 software (OriginLab Corporation, Northampton, MA, USA). All experiments were performed in triplicate (*n* = 3), and experimental results were expressed as the mean ± standard deviation.

## 3. Results and Discussion

### 3.1. Determination of Carbonyl Content

During meat processing, free radicals can attack the free amine (ε-NH2) groups and imino groups from the amino acid side chain, e.g., arginine, lysine, threonine, and proline, by abstracting a hydrogen atom from the neighboring carbon, leading to the formation of a carbon-centered protein radical. In a further step, oxidized forms of the metal ions would accept the lone electron of the carbon radical to form an imino group which is spontaneously hydrolyzed to yield the corresponding aldehyde moiety—that is, carbonyl groups [23]. As a common index reflecting the extent of protein oxidation, carbonyl content has a strong correlation with protein functionalities, e.g., texture, water-holding capacity, and nutritional value [20].

Compared with the non-oxidized MPs (blank)**,** an obvious increment in carbonyl content was observed in the oxidized MPs in the absence of HYP (Table 1). This was in agreement with the study of Wang et al. [24], which also showed that the amine groups on the side chains of MPs were attacked by radicals resulting in the increment in carbonyl content. As for oxidized treatments, the carbonyl content of samples was decreased (*p* < 0.05) with the decrement in NaCl from 0.6 M to 0.2 M in the absence or presence of HYP. This might be ascribed to the fact that some active amine groups were buried in MPs because of MPs aggregation at low salt concentration; thus, fewer amine groups were attacked by hydroxyl radicals [25,26]. Additionally, an obvious decrement in the carbonyl content was shown with the incorporation of HYP (*p* < 0.05). Moreover, the carbonyl content was decreased with the increased dose of HYP. The carbonyl content of MPs was reduced (*p* < 0.05) by 13.47%, 27.76%, and 43.26% at 0.2 M NaCl, and reduced by 9.24%, 48.18%, and 63.37% at 0.6 M NaCl in the presence of 10, 50, and 250 μM/g HYP, respectively. The results were consistent with previous studies, which indicated that polyphenols decreased the carbonyl content of MPs [6,27].

### 3.2. Determination of Free Amines

The free amine levels of different groups are shown in Table 1. As expected, the oxidized MPs had an obviously lower (*p* < 0.05) free amines content than the blank. This is in agreement with the study of Li et al. [28], who reported that the level of free amine groups in oxidized MPs was obviously reduced compared with that in the non-oxidized MPs from the large yellow croaker. In addition, the level of free amine groups in oxidized MPs was significantly decreased (*p* < 0.05) with the increasing NaCl concentration. At higher salt concentrations, MPs has a higher solubility with more soluble MPs [24,29]. Therefore, the amine groups of amino acid residues on the MPs surface are more easily attacked by free radicals. As for HYP treatments, it was notably shown that HYP decreased the loss of free amine groups in a dose-dependent manner. The content of free amines was increased (*p* < 0.05) by 4.24%, 10.67%, and 15.27% at 0.2 M NaCl, and by 3.45%, 7.81%, and 14.23% at 0.6 M NaCl in the presence of 10, 50, and 250 μM/g HYP, coinciding with their decreased carbonyl content, respectively. The results are in agreement with the study of Pan et al. [30], which showed that the addition of 5, 25, and 125 μM/g protein gallic acid protected the free amines of MPs from oxidation. Nevertheless, this is different from the previous study, which showed that chlorogenic acid could not inhibit the hydroxyl radical-mediated free amine loss at low or middle doses but induced polyphenolic-initiated free amine losses at a high dose [27]. Compared with chlorogenic acid, HYP has more phenol hydroxyl groups, which would show a higher antioxidative activity and reduce the loss of free amine groups under oxidation, particularly for a relatively low concentration of HYP-adding groups. For 250 μM/g HYP treatment, the formation of more stable protein-bound phenoxyl radicals might further reduce the oxidation-mediated loss of free amine groups. It was inferred that the formation of a covalent adduction of quinone between free amines and polyphenolics would accelerate the loss of free amines at a high level of polyphenols upon oxidation [24,31,32]. The high-dose HYP did not show such a polyphenolic-initiated loss of free amine groups, suggesting that the pathway for HYP to form amine–quinone adducts might not happen.

### 3.3. Determination of SH Content

MPs are abundant in sulfhydryl groups, which are highly sensitive and vulnerable to attack by free radicals, resulting in the formation of inter- and intramolecular disulfide bond linkages [33,34]. Compared with the blank, the SH content of oxidized MPs was reduced (*p* < 0.05) by 20.43%. Upon oxidation, the SH content of oxidized MPs was significantly decreased (*p* < 0.05) with the increasing NaCl concentration. This was in line with the changes in carbonyls. At a higher salt concentration, MPs are in a swollen state with more soluble MPs [24,26]. As a consequence, more sulfhydryl groups of cysteine residues were exposed to the MPs surface and vulnerable to attack by hydroxyl radicals to form S-S bonds [35]. Furthermore, the incorporation of HYP caused a sharp and significant reduction in the SH content of oxidized MPs. At 0.2 M NaCl, the SH content of MPs was decreased (*p* < 0.05) from 51.57 nmol/mg protein to 43.79, 38.99, and 31.42 nmol/mg protein in the presence of 10, 50, and 250 μM/g HYP, respectively. At 0.6 M NaCl, the SH content of MPs was decreased (*p* < 0.05) from 46.47 nmol/mg protein to 36.65, 31.55, and 24.24 nmol/mg protein in the presence of 10, 50, and 250 μM/g HYP, respectively. These results might be ascribed to the formation of sulfhydryl–quinone adducts between the quinone of HYP and SH of MPs by Michael addition [36,37]. For example, many phyto-phenolic substances, such as quercetin [38], rutin [39], gallic acid [40], and EGCG [41] could decrease the SH content of meat protein via additive reactions between the SH of MPs and the quinone of polyphenolics.

### 3.4. Determination of Surface Hydrophobicity

**Figure 1:**
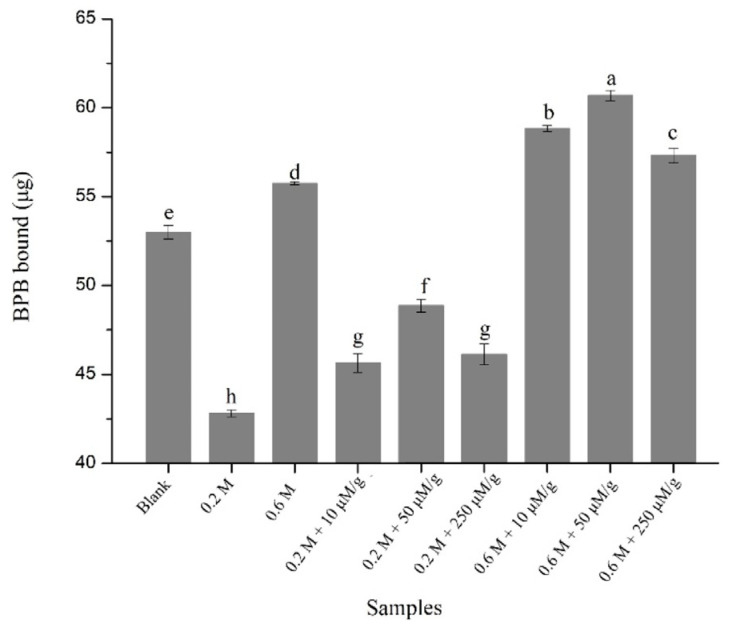
Surface hydrophobicity of the non-oxidized MPs (blank) and oxidized MPs prepared with HYP (10, 50, and 250 μM/g) at different NaCl concentrations (0.2 and 0.6 M). Values are means ± SD. Different letters indicate significant differences (*p* < 0.05) (*n* = 3).

The surface hydrophobicity of MPs may reflect the extent of unfolding of MPs and is a considerable molecular characteristic associated with MPs functionality [17]. The effects of HYP on the surface hydrophobicity of the samples were examined (Figure 1). Oxidation obviously improved (*p* < 0.05) the surface hydrophobicity of MPs. As for NaCl concentration, the surface hydrophobicity of MPs was obviously increased (*p* < 0.05) with the increment in NaCl concentration, which might be owing to the high solubility of MPs at high NaCl concentrations [42,43]. In addition, an obvious increase in surface hydrophobicity was shown in the presence of 10, 50, and 250 μM/g HYP regardless of salt concentration. At 0.2 M NaCl, the surface hydrophobicity was increased (*p* < 0.05) by 6.56%, 14.08%, and 7.68% in the presence of 10, 50, and 250 μM/g HYP, respectively. At 0.6 M NaCl, the surface hydrophobicity was increased (*p* < 0.05) by 5.54%, 8.86%, and 2.80% in the presence of 10, 50, and 250 μM/g HYP, respectively. This is consistent with the results of the study by Jia et al. [39], which showed an increment in surface hydrophobicity with the addition of rutin. The results indicated that HYP caused the exposure of masked hydrophobic groups, which might permit more functional groups to become involved in the thermal gelation [27]. Nevertheless, compared with sample treated with 50 μM/g HYP, 250 μM/g HYP caused a decrease (*p* < 0.05) in surface hydrophobicity. The higher dose of HYP might increase the extent of MPs unfolding and expose more hydrophobic groups, which might facilitate the formation of hydrophobic aggregates by hydrophobic interactions between MPs and make the hydrophobic groups unavailable for bromophenol blue. Furthermore, the non-covalent hydrophobic interactions between HYP and the exposed hydrophobic groups might also mask some hydrophobic groups [44].

### 3.5. Determination of Gel Strength and WHC

Oxidation could induce structural changes in MPs and concomitant changes in functional properties. The effect of HYP on the gel strength and WHC was shown in Table 2. Oxidation caused a significant decrease in gel strength (*p* < 0.05), which might be result from the loss of SH content. The reduction of sulfhydryl groups might lead to a decrease in disulfide bond formation during heating, thus reducing the interactions between MPs and MPs and preventing the formation of a fine three-dimensional gel network [17]. Upon oxidation, an obvious increase in gel strength was shown with the increment in NaCl concentration (*p* < 0.05). As mentioned earlier, MPs have a higher solubility with higher amounts of soluble MPs at high NaCl concentrations, facilitating the formation of ordered intra- and intermolecular crosslinks within MPs [45]. The addition of HYP caused a significant increase in gel strength regardless of salt concentration. Moreover, the gel strength was increased with increasing HYP concentration. The gel strength was increased (*p* < 0.05) by 42.42%, 81.82%, and 127.27% at 0.2 M NaCl, and by 44.44%, 66.67%, and 100% at 0.6 M NaCl in the presence of 10, 50, and 250 μM/g HYP, indicating the formation of a stronger gel network. On one hand, the addition of HYP caused the exposure of masked hydrophobic groups of MPs, reinforcing the MPs–MPs interaction and subsequently facilitating the formation of an MPs gel network. On the other hand, the MPs–HYP crosslinks enhanced by the formation of thiol–quinone adducts might also facilitate the formation of an MPs gel network. This was similar to the results of research by Jia et al. [39], which ascribed the increased strength of MPs gel to the enhanced rutin–protein cross-linking by the formation of thiol–quinone adducts.

The variation trend in WHC was in accordance with gel strength. The WHC of MPs gel was significantly decreased (*p* < 0.05) after oxidation. This is in agreement with previous research [46], in which a significant oxidation-induced decrease in WHC was shown to be likely due to the formation of an inferior gel network. Regarding NaCl concentration, the WHC of an oxidized MPs gel was increased with the increasing NaCl concentration (*p* < 0.05), which might be owing to the higher solubility of MPs and enhanced environmental ionic force at higher salt concentrations [47]. Moreover, the incorporation of HYP caused a dose-dependent increment in WHC. The WHC was increased (*p* < 0.05) by 9.92%, 34.89%, and 50.90% at 0.2 M NaCl, and by 7.57%, 15.71%, and 27.11% at 0.6 M NaCl in the presence of 10, 50, and 250 μM/g HYP, respectively. Notably, the WHC of an MPs gel treated with 250 μM/g HYP at NaCl was higher than the non-oxidized MPs gel. As mentioned above, the addition of HYP promoted the ordered crosslinks of MPs–MPs and MPs–HYP during gelation, resulting in a better WHC of the MPs gel.

### 3.6. Microstructure of MPs Gel

**Figure 2:**
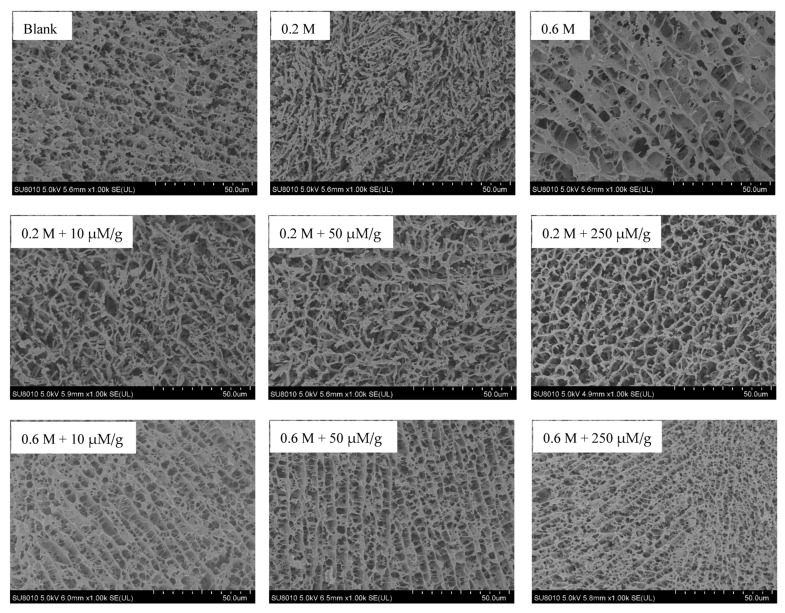
Cryo-SEM images of the non-oxidized MPs gel (blank) and oxidized MPs gels prepared with HYP (10, 50, and 250 μM/g) at different NaCl concentrations (0.2 and 0.6 M). Scale bars indicate 50.0 μm.

The microstructure images of different MPs gels are shown in Figure 2. The blank sample presented a homogeneous and compact network structure. In contrast, the oxidized MPs gel at 0.6 M NaCl showed a loose lamellar structure with more and larger pores, suggesting that oxidation induced the destruction of gel structure. This is consistent with the research of Guo et al. [17], which showed that the gel structure of an oxidized MPs gel at 0.6 M NaCl was rough and cracked.

In agreement with previous research [48], the oxidized MPs gels at 0.2 M NaCl presented coarse cross-linked filament structures, which would apparently result in the poor WHC and texture. The incorporation of HYP caused the formation of more orderly crosslinks via fibrous filaments at 0.2 M NaCl and more regular and lamellar structure with smaller and more homogeneous pores at 0.6 M NaCl, resulting in the better gel quality. As mentioned above, the incorporation of HYP caused the exposure of buried hydrophobic groups of MPs and further formation of thiol–quinone adducts, reinforcing the ordered crosslinks of MPs–MPs and MPs–HYP during thermal gelation, facilitating the formation of a better network structure, ultimately resulting in the enhanced gel strength and WHC.

## 4. Conclusions

The present work suggested that HYP might prevent oxidative damage to the physicochemical characteristics of MPs and the corresponding gel quality in a dose-dependent manner, at both 0.2 M and 0.6 M NaCl concentrations. Of special note, HYP reduced carbonyl content and decreased the loss of free amine groups. The total sulfhydryl content was decreased, possibly resulting from the formation of thiol–quinone adducts via Michael addition. The surface hydrophobicity was significantly increased. Nevertheless, the highest dose of HYP caused a decrease in surface hydrophobicity, possibly due to the increased extent of MPs unfolding and the concomitant aggregation of MPs by hydrophobic interaction. Furthermore, gel strength and WHC were increased, which might result from the formation of more ordered crosslinks of MPs–MPs and MPs–HYP during thermal gelation. Consequently, HYP can protect against the oxidative damage of MPs. These results may provide a reference for the application of natural antioxidants containing HYP in meat products.

## Figures and Tables

**Table 1:** Physicochemical characteristics of non-oxidized MPs (blank) and oxidized MPs prepared with HYP (10, 50, and 250 μM/g) at different NaCl concentrations (0.2 and 0.6 M).

Samples	Carbonyl (nmol/mg Protein)	Free Amines (nmol/mg Protein)	SH (nmol/mg Protein)
Blank	0.33 ± 0.19 i	80.15 ± 2.98 a	58.40 ± 0.98 a
0.2 M NaCl	2.45 ± 0.11 c	56.63 ± 1.29 d	51.57 ± 0.64 b
0.6 M NaCl	3.03 ± 0.08 a	51.87 ± 2.13 e	46.47 ± 0.61 c
0.2 M NaCl + 10 μM/g HYP	2.12 ± 0.08 d	59.03 ± 1.42 c	43.79 ± 0.33 d
0.2 M NaCl + 50 μM/g HYP	1.77 ± 0.12 e	62.67 ± 1.38 b	38.99 ± 0.84 e
0.2 M NaCl + 250 μM/g HYP	1.39 ± 0.04 g	65.28 ± 2.03 b	31.42 ± 0.98 f
0.6 M NaCl + 10 μM/g HYP	2.75 ± 0.12 b	53.66 ± 2.03 de	36.65 ± 1.51 e
0.6 M NaCl + 50 μM/g HYP	1.57 ± 0.16 f	55.92 ± 1.67 d	31.55 ± 2.01 f
0.6 M NaCl + 250 μM/g HYP	1.11 ± 0.04 h	59.25 ± 2.04 c	24.24 ± 1.50 g

Note: Values are the mean of triplicate values ± SD. Different lowercase letters in the same column indicate a significant difference (*p* < 0.05).

**Table 2:** Gel strength and WHC of non-oxidized MPs gel (blank) and oxidized MPs gels prepared with HYP (10, 50, and 250 μM/g) at different NaCl concentrations (0.2 and 0.6 M).

Samples	Gel Strength (g·mm)	WHC (%)
Blank	0.25 ± 0.010 c	67.14 ± 1.56 b
0.2 M NaCl	0.066 ± 0.013 h	27.23 ± 1.33 g
0.6 M NaCl	0.18 ± 0.012 d	59.72 ± 0.29 d
0.2 M NaCl + 10 μM/g HYP	0.094 ± 0.013 g	29.93 ± 0.24 g
0.2 M NaCl + 50 μM/g HYP	0.12 ± 0.0065 f	36.73 ± 0.65 f
0.2 M NaCl + 250 μM/g HYP	0.15 ± 0.011 e	41.09 ± 3.43 e
0.6 M NaCl + 10 μM/g HYP	0.26 ± 0.013 c	64.24 ± 2.21 c
0.6 M NaCl + 50 μM/g HYP	0.30 ± 0.019 b	69.10 ± 1.15 b
0.6 M NaCl + 250 μM/g HYP	0.36 ± 0.014 a	75.91 ± 2.12 a

Note: Values are the mean of triplicate values ± SD. Different lowercase letters in the same column indicate significant differences (*p* < 0.05).

## Data Availability

The data that support the findings of this study are available on request from the corresponding author.
